# Supplementary material for: Feasibility and Validity of a Low-Cost Racing Simulator in Driving Assessment after Stroke
Source: Geriatrics (Basel). 2020 May 29;5(2):35. doi: 10.3390/geriatrics5020035 (PMC7345038; doi:10.3390/geriatrics5020035)
Supplement: Supplementary file 1 [file geriatrics-05-00035-s001.pdf]

Table S1. Components, and their prices, used in consumer−grade racing simulator setup.

Item	Component Used	Minimum Simulator Requirement	Cost, USD
Consumer−grade personal computer			
Case	Corsair Graphite Series 780T Full Tower Windowed Case	varies	$144
Power supply	Corsair RM Series RM850	varies	$150
Processor	Intel Core i7 processor	AMD Athlon X2 2.8 GHz, Intel Core 2 Duo 2.4 GHz	$350
Hard drive	Samsung 850 EVO solid state drive	15GB space	$100
Memory	EVGA 16GB DDR3 2400 RAM	2GB RAM	$80
Motherboard	ASUS Z107−A (ATX)	Varies	$130
Graphics card	nVidia GeForce GTX1070	DirectX 10.1 compatible (e.g., Nvidia GeForce GT 460)	$400
Mouse	Standard USB optical mouse	n/a	n/a
Keyboard	Standard USB keyboard	n/a	n/a
Display & Controls			
Television	40″ Class LED 1080p HDTV	Any compatible display	$200
HD cable	High-quality generic HD cable	n/a	$50
Steering wheel	Thrustmaster T500RS	Any compatible controller	$600
Software			
OS	Microsoft Windows 10, 64-bit edition	Windows 7 Sp1–10	$140
Simulator	Assetto Corsa	n/a	$45
Total racing simulator setup cost (not including keyboard and mouse)			$2389

GHZ = Gigahertz, GB = Gigabyte, DDR3 = Double Data Rate 3rd generation, RAM = random access memory, ATX = Advanced Technology eXtended, HD = high definition, OS = operating system.

Table S2. Comparison of research simulator and racing simulator outcomes for participants using foot affected by stroke vs. foot unaffected by stroke to operate driving pedal, using chi-squared test or independent samples *t*-test.

Research Simulator Outcomes							
**Independent samples *t*-test**						**95% CI of the difference**	
	**F**	**Sig.**	**t**	**df**	**Sig. (2-tailed)**	**Lower**	**Upper**
Scenario 1 SDLP	1.022	0.323	−0.666	22	0.513	−1.260	0.648
Scenario 2 mean speed	0.002	0.963	1.384	24	0.179	−2.917	14.786
Scenario 2 max speed	0.03	0.863	0.363	24	0.72	−6.310	8.999
Scenario 3 mean speed	0.116	0.736	1.468	24	0.155	−2.544	15.076
Scenario 4 TTC	0.936	0.343	−0.868	24	0.394	−0.275	0.112
**Chi−square test**							
				**N**	**Value**	**df**	**p**
Scenario 2 collision	*(Sample too small)*			26	0.042	1	0.84
Scenario 2 pedal error	*(Sample too small)*			24	0	1	1
Scenario 2 excursion	*(Sample too small)*				1.242a	1	0.27

Racing Simulator Outcomes							
**Independent samples *t*-test**						**95% CI of the difference**	
	**F**	**Sig.**	**t**	**df**	**Sig. (2-tailed)**	**Lower**	**Upper**
Preferred foot pedal reaction time			−1.85	10	0.094	−2.119	0.196

Silverstone lap 1 time	0.163	0.69	−0.939	23	0.357	−81.298	30.517
Silverstone lap 2 time	0.006	0.938	−0.71	22	0.485	−54.387	26.631
Silverstone lap time delta	2.762	0.111	1.089	22	0.288	−19.413	62.344
Silverstone lap 1 max # tires off track	0.842	0.368	0.429	23	0.672	−1.214	1.848
Silverstone lap 2 max # tires off track	1.103	0.304	0.465	23	0.646	−1.423	2.249
Silverstone total max # tires off track	0.168	0.685	0.525	23	0.604	−2.145	3.605
Monza lap 1 time	4.494	0.046	−2.265	20.2	0.035	−81.404	−3.386
Monza lap 2 time	0.628	0.437	−1.174	20	0.254	−126.575	35.403
Monza lap time delta	1.308	0.266	0.036	20	0.972	−73.520	76.109
Monza lap 1 max # tires off track	2.239	0.149	0.634	22	0.532	−1.123	2.112
Monza lap 2 max # tires off track	0.977	0.335	0.871	20	0.394	−1.317	3.206
Monza total max # tires off track	0.149	0.703	0.84	20	0.411	−2.059	4.836

SDLP = standard deviation of lap position, MPH = miles per hour, TTC = time−to-collision.

Table S3. Complete Spearman correlations of clinical psychometric and motor testing to research and racing simulator outcomes.

Clinical Test	r_S	*p*
Trail Making Test Part A		
Highway scenario SDLP	−0.094	$p = 0.58$
Rural scenario mean speed	0.151	$p = 0.35$
Rural scenario max speed	−0.087	$p = 0.59$
Rural scenario passing reaction time	0.300	$p = 0.06$
Construction scenario mean speed	−0.032	$p = 0.85$
Urban scenario TTC	−0.117	$p = 0.47$
Silverstone lap 1 time	0.233	$p = 0.15$
Silverstone lap 2 time	0.346*	$p = 0.04$
Silverstone lap 2 max # tires off track	0.079	$p = 0.64$
Silverstone total max # tires off track	0.096	$p = 0.57$
Monza lap 1 time	0.201	$p = 0.21$
Monza lap 2 time	0.452**	$p < 0.01$
Monza lap 2 max # tires off track	0.094	$p = 0.58$
Monza total max # tires off track	0.161	$p = 0.35$
Drag race preferred foot pedal reaction time	−0.104	$p = 0.65$
Snellgrove Maze Task		
Highway scenario SDLP	0.241	$p = 0.15$
Rural scenario mean speed	0.158	$p = 0.33$
Rural scenario max speed	0.132	$p = 0.42$
Rural scenario passing reaction time	0.337*	$p = 0.03$
Construction scenario mean speed	−0.040	$p = 0.80$
Urban scenario TTC	−0.110	$p = 0.50$
Silverstone lap 1 time	0.175	$p = 0.29$
Silverstone lap 2 time	0.307	$p = 0.07$
Silverstone lap 2 max # tires off track	−0.048	$p = 0.78$
Silverstone total max # tires off track	−0.195	$p = 0.24$
Monza lap 1 time	0.178	$p = 0.29$
Monza lap 2 time	0.245	$p = 0.15$

Monza lap 2 max # tires off track	−0.057	$p = 0.74$
Monza total max # tires off track	−0.129	$p = 0.45$
Drag race preferred foot pedal reaction time	0.423	$p = 0.06$
Rapid Pace Walk		
Highway scenario SDLP	−0.077	$p = 0.65$
Rural scenario mean speed	0.284	$p = 0.08$
Rural scenario max speed	0.149	$p = 0.37$
Rural scenario passing reaction time	0.049	$p = 0.77$
Construction scenario mean speed	0.031	$p = 0.85$
Urban scenario TTC	−0.085	$p = 0.61$
Silverstone lap 1 time	0.236	$p = 0.15$
Silverstone lap 2 time	0.275	$p = 0.11$
Silverstone lap 2 max # tires off track	0.097	$p = 0.57$
Silverstone total max # tires off track	0.160	$p = 0.35$
Monza lap 1 time	−0.018	$p = 0.92$
Monza lap 2 time	0.394*	$p = 0.02$
Monza lap 2 max # tires off track	0.259	$p = 0.13$
Monza total max # tires off track	0.232	$p = 0.18$
Drag race preferred foot pedal reaction time	0.305	$p = 0.18$
Motricity Index, left side		
Highway scenario SDLP	0.061	$p = 0.72$
Rural scenario mean speed	−0.131	$p = 0.42$
Rural scenario max speed	−0.025	$p = 0.88$
Rural scenario passing reaction time	−0.144	$p = 0.37$
Construction scenario mean speed	0.079	$p = 0.63$
Urban scenario TTC	0.049	$p = 0.76$
Silverstone lap 1 time	−0.158	$p = 0.38$
Silverstone lap 2 time	−0.248	$p = 0.14$
Silverstone lap 2 max # tires off track	−0.070	$p = 0.68$
Silverstone total max # tires off track	−0.076	$p = 0.65$
Monza lap 1 time	−0.005	$p = 0.98$
Monza lap 2 time	−0.113	$p = 0.51$
Monza lap 2 max # tires off track	0.208	$p = 0.22$
Monza total max # tires off track	0.226	$p = 0.19$
Drag race preferred foot pedal reaction time	−0.339	$p = 0.13$
Motricity Index, right side		
Highway scenario SDLP	0.075	$p = 0.66$
Rural scenario mean speed	−0.476**	$p < 0.01$
Rural scenario max speed	−0.352*	$p = 0.03$
Rural scenario passing reaction time	−0.588**	$p < 0.01$
Construction scenario mean speed	−0.460**	$p < 0.01$
Urban scenario TTC	0.179	$p = 0.28$
Silverstone lap 1 time	−0.286	$p = 0.08$
Silverstone lap 2 time	−0.094	$p = 0.58$
Silverstone lap 2 max # tires off track	−0.325*	$p < 0.05$
Silverstone total max # tires off track	−0.357*	$p = 0.03$
Monza lap 1 time	0.188	$p = 0.26$
Monza lap 2 time	0.044	$p = 0.80$
Monza lap 2 max # tires off track	0.018	$p = 0.92$
Monza total max # tires off track	−0.008	$p = 0.96$
Drag race preferred foot pedal reaction time	0.077	$p = 0.74$
Jamar grip strength, left side		
Highway scenario SDLP	0.132	$p = 0.43$
Rural scenario mean speed	0.052	$p = 0.75$
Rural scenario max speed	0.075	$p = 0.65$
Rural scenario passing reaction time	0.118	$p = 0.47$
Construction scenario mean speed	0.199	$p = 0.22$
Urban scenario TTC	−0.126	$p = 0.44$

Silverstone lap 1 time	0.018	$p = 0.91$
Silverstone lap 2 time	−0.167	$p = 0.32$
Silverstone lap 2 max # tires off track	0.011	$p = 0.95$
Silverstone total max # tires off track	0.157	$p = 0.35$
Monza lap 1 time	−0.258	$p = 0.12$
Monza lap 2 time	−0.106	$p = 0.54$
Monza lap 2 max # tires off track	0.343*	$p = 0.04$
Monza total max # tires off track	0.374*	$p = 0.03$
Drag race preferred foot pedal reaction time	−0.460*	$p = 0.04$
Jamar grip strength, right side		
Highway scenario SDLP	0.150	$p = 0.39$
Rural scenario mean speed	−0.170	$p = 0.31$
Rural scenario max speed	−0.057	$p = 0.74$
Rural scenario passing reaction time	−0.305	$p = 0.07$
Construction scenario mean speed	−0.209	$p = 0.21$
Urban scenario TTC	0.114	$p = 0.50$
Silverstone lap 1 time	−0.208	$p = 0.22$
Silverstone lap 2 time	−0.098	$p = 0.58$
Silverstone lap 2 max # tires off track	−0.127	$p = 0.47$
Silverstone total max # tires off track	−0.128	$p = 0.47$
Monza lap 1 time	−0.028	$p = 0.87$
Monza lap 2 time	0.088	$p = 0.62$
Monza lap 2 max # tires off track	0.132	$p = 0.46$
Monza total max # tires off track	0.121	$p = 0.50$
Drag race preferred foot pedal reaction time	0.181	$p = 0.43$

SDLP = standard deviation of lap position, TTC = time−to−collision.

*$p < 0.05$; **$p < 0.01$.

Table S4. Complete Spearman correlations of racing simulator to research simulator outcomes.

**Racing Simulator Variable**	**r_S**	***p***
Silverstone lap 1 time		
Highway scenario SDLP	−0.228	$p = 0.18$
Rural scenario mean speed	−0.190	$p = 0.25$
Rural scenario max speed	−0.474**	$p < 0.01$
Rural scenario passing reaction time	0.120	$p < 0.466$
Construction scenario mean speed	−0.336*	$p = 0.04$
Urban scenario TTC	0.231	$p = 0.16$
Silverstone lap 2 time		
Highway scenario SDLP	0.035	$p = 0.84$
Rural scenario mean speed	−0.219	$p = 0.19$
Rural scenario max speed	−0.529**	$p < 0.01$
Rural scenario passing reaction time	0.025	$p = 0.83$
Construction scenario mean speed	−0.417*	$p = 0.01$
Urban scenario TTC	0.289	$p = 0.08$
Silverstone lap 2 max # tires off track		
Highway scenario SDLP	0.274	$p = 0.112$
Rural scenario mean speed	0.521**	$p < 0.01$
Rural scenario max speed	0.454**	$p < 0.01$
Rural scenario passing reaction time	0.424**	$p < 0.01$
Construction scenario mean speed	0.340*	$p = 0.04$
Urban scenario TTC	−0.017	$p = 0.92$
Silverstone total max # tires off track		
Highway scenario SDLP	0.114	$p = 0.51$
Rural scenario mean speed	0.396*	$p = 0.01$
Rural scenario max speed	0.311	$p = 0.06$
Rural scenario passing reaction time	0.272	$p = 0.10$
Construction scenario mean speed	0.280	$p = 0.09$

Urban scenario TTC	−0.082	$p = 0.62$
Monza lap 1 time		
Highway scenario SDLP	−0.126	$p = 0.47$
Rural scenario mean speed	−0.379*	$p = 0.02$
Rural scenario max speed	−0.604**	$p < 0.01$
Rural scenario passing reaction time	0.023	$p = 0.89$
Construction scenario mean speed	−0.611**	$p < 0.01$
Urban scenario TTC	0.728**	$p < 0.01$
Monza lap 2 time		
Highway scenario SDLP	−0.138	$p = 0.44$
Rural scenario mean speed	−0.265	$p = 0.12$
Rural scenario max speed	−0.490**	$p < 0.01$
Rural scenario passing reaction time	−0.082	$p = 0.64$
Construction scenario mean speed	−0.492**	$p < 0.01$
Urban scenario TTC	0.495**	$p < 0.01$
Monza lap 2 max # tires off track		
Highway scenario SDLP	0.251	$p = 0.16$
Rural scenario mean speed	0.092	$p = 0.59$
Rural scenario max speed	0.149	$p = 0.39$
Rural scenario passing reaction time	−0.100	$p = 0.56$
Construction scenario mean speed	0.118	$p = 0.49$
Urban scenario TTC	−0.006	$p = 0.97$
Monza total max # tires off track		
Highway scenario SDLP	0.298	$p = 0.09$
Rural scenario mean speed	0.305	$p = 0.07$
Rural scenario max speed	0.335*	$p < 0.05$
Rural scenario passing reaction time	−0.084	$p = 0.63$
Construction scenario mean speed	0.247	$p = 0.15$
Urban scenario TTC	−0.202	$p = 0.24$
Drag race preferred foot pedal reaction time		
Highway scenario SDLP	0.440*	$p < 0.05$
Rural scenario mean speed	0.062	$p = 0.79$
Rural scenario max speed	−0.021	$p = 0.93$
Rural scenario passing reaction time	0.248	$p = 0.28$
Construction scenario mean speed	−0.356	$p = 0.11$
Urban scenario TTC	0.290	$p = 0.20$

SDLP = standard deviation of lap position, TTC = time−to−collision. *$p < 0.05$; **$p < 0.01$.

Table S5. Research simulator variables in the binary logistic regression Model 1b (with outliers replaced).

Low− Versus High−Risk Fitness−to−Drive Screen									
								95% CI	
		B	SE	Wald	df	Sig.	Exp(B)	Lower	Upper
Step 1	Scenario 1 SDLP	−8.988	7.835	1.316	1	0.251	0.000	0.000	582.735
	Scenario 2 maximum speed (MPH)	−.122	0.098	1.555	1	0.212	0.885	0.731	1.072
	Scenario 2 reaction time (sec)	1.532	0.909	2.839	1	0.092	4.628	0.779	27.498
	Scenario 2 road excursion (y/n)	2.602	8.262	0.099	1	0.753	13.488	–––	–––
	Scenario 2 collision (y/n)	0.340	8.001	0.002	1	0.966	1.404	–––	–––
	Scenario 4 TTC	−15.202	9.943	2.338	1	0.126	0.000	0.000	72.640
	Constant	13.741	13.236	1.078	1	0.299	994046.145		–––

SDLP = standard deviation of lap position, MPH = miles per hour, TTC = time−to−collision.

Table S6. Racing simulator variables in the binary logistic regression (outliers replaced) Model 2b.

**Low-Versus High-Risk Fitness-to-Drive Screen**									
								95% CI	
		B	SE	Wald	df	Sig.	Exp(B)	Lower	Upper
Step 1	Monza Lap 2 Time (sec)	0.028	0.02	1.77	1	0.18	1.028	0.987	1.071
	Silverstone Lap 2 Time (sec)	0.01	0.02	0.187	1	0.67	1.010	0.965	1.057
	Monza Total Max Tires Off Track	−0.047	0.276	0.029	1	0.87	0.954	0.555	1.640
	Silverstone Total Max Tires Off Track	−0.242	0.246	0.967	1	0.33	0.785	0.485	1.271
	Constant	−9.60	4.27	5.05	1	0.03	0.000		
